# Basement membrane-related regulators for prediction of prognoses and responses to diverse therapies in hepatocellular carcinoma

**DOI:** 10.1186/s12920-023-01504-z

**Published:** 2023-04-20

**Authors:** Ruili Ding, Chuanbing Zhao, Yixin Jing, Rong Chen, Qingtao Meng

**Affiliations:** 1 Department of Anesthesiology, Renmin Hospital of Wuhan University, No.238, Jiefang Road, Wuhan, 430061 Hubei Province China; 2 Department of Pancreatic Surgery, Renmin Hospital of Wuhan University, No.238, Jiefang Road, Wuhan, 430061 Hubei Province China

**Keywords:** Hepatocellular carcinoma, BMR, Basement membrane, Drug sensitivity, Immunotherapy

## Abstract

**Background:**

Hepatocellular carcinoma (HCC) remains a global health threat. Finding a novel biomarker for assessing the prognosis and new therapeutic targets is vital to treating this patient population. Our study aimed to explore the contribution of basement membrane-related regulators (BMR) to prognostic assessment and therapeutic response prediction in HCC.

**Material and methods:**

The RNA sequencing and clinical information of HCC were downloaded from TCGA-LIHC, ICGC-JP, GSE14520, GSE104580, and CCLE datasets. The BMR signature was created by the Least Absolute Shrinkage and Selection Operator (LASSO) algorithm and used to separate HCC patients into low- and high-risk groups. We conducted analyses using various R 4.1.3 software packages to compare prognoses and responses to immunotherapy, transcatheter arterial chemoembolization (TACE), and chemotherapeutic drugs between the groups. Additionally, stemness indices, molecular functions, and somatic mutation analyses were further explored in these subgroups.

**Results:**

The BMR signature included 3 basement membrane-related genes (CTSA, P3H1, and ADAM9). We revealed that BMR signature was an independent risk contributor to poor prognosis in HCC, and high-risk group patients presented shorter overall survival. We discovered that patients in the high-risk group might be responsive to immunotherapy, while patients in the low-risk group may be susceptible to TACE therapy. Over 300 agents were screened to identify effective drugs for the two subgroups.

**Conclusion:**

Overall, basement membrane-related regulators represent novel biomarkers in HCC for assessing prognosis, response to immunotherapy, the effectiveness of TACE therapy, and drug susceptibility.

**Supplementary Information:**

The online version contains supplementary material available at 10.1186/s12920-023-01504-z.

## Introduction

Hepatocellular carcinoma (HCC) continues to be a global health threat, with  incidence rates increasing worldwide [1, 2]. By 2025, the number of new cases of HCC is anticipated to exceed 1,000,000 per year [3]. Despite the advent of novel therapeutic strategies, including immunotherapy and targeted therapies, the outcomes of HCC remain unsatisfactory [4–6]. Therefore, discovering new targets or identifying new biomarkers for treating this patient population is a high priority for researchers in this field.

The basement membrane (BM) is an extracellular matrix located beneath the epithelial cells of animals [7], of which collagen, laminin, and integrins are the main components. It is now understood that in malignant tumors of epithelial origin, tumor cells pass through the BM before further invasion and metastasis. Collagen, laminin, and integrins play a crucial role in cancer development by contributing to essential stages of oncogenesis, such as proliferation, apoptosis, angiogenesis, invasion, and metastasis [8–12]. Current evidence suggests that laminin-5 (LN5), a component of BM, is associated with a highly metastatic phenotype of HCC and can be utilized as a diagnostic indicator to identify metastases in adjacent tumor tissues [13]. Moreover, extracellular matrix protein I has been found to promote tumor progression in HCC after heat treatment [14]. The recent literature has suggested that BM might be a prognostic marker and target for HCC patients [15, 16]. The above studies overlap in their assertion that BM has enormous prospects for treating HCC. 

Despite extensive research on the basement membrane in recent years, most studies have been limited to the morphological level. Therefore, it is essential to explore the function of basement membrane-associated genes in HCC from a gene regulation perspective. For instance, LAMA4 is reportedly highly expressed in HCC patients and could be a promising target for HCC treatment [17]. Recent studies have also defined a range of basement membrane-associated genes [18]. To our knowledge, no study has hitherto explored the role of these BM-related genes in HCC. Therefore, it is essential to examine the association between BM-related clusters and the prognoses and immune profiles of patients with HCC.

Herein, we revealed that the basement membrane-related regulators (BMR) played favorable roles in determining prognoses, immunotherapy responses, and drug sensitivity in HCC patients. 

## Materials and methods

### Data acquisition and procession

The RNA sequencing and clinical information of patients with HCC were downloaded from The Cancer Genome Atlas Liver Hepatocellular Carcinoma (TCGA-LIHC) (https://portal.gdc.cancer.gov/), International Cancer Genome Consortium (IGCG)-JP (https://dcc.icgc.org/) and Gene Expression Omnibus (GEO) (https∶/http://ww.ncbinlm.nih.gov/geo/) (GSE14520 and GSE104580) databases. In addition, a list of BM-related genes was extracted from existing publications [18]. The somatic mutation files of HCC patients were acquired from the Genomic Data Commons (GDC) database. Moreover, the mRNA expression data of HCC cell lines were retrieved from Cancer Cell Line Encyclopedia (CCLE) database [19]. We applied the "limma" [20] and "sva" [21] packages to normalize the expression data originating from the GEO database. 

### Prognostic analyses

#### Date Processing before construction of the BMR signature

We first utilized the "limma" [20] package (|logFC)|≥ 1 and FDR < 0.05) to filter differentially expressed genes (DEGs) in HCC tissue versus liver tissue from the list of basement membrane-associated genes. The obtained DEGs underwent univariate Cox regression analysis to identify genes associated with prognosis (adjusted P-value<0.05), regarded as candidate genes. Subsequently, we employed "igraph" [22], "psych", "reshape2" [23], and "RColorBrewer" packages to construct a correlation network of these candidate genes.

The unsupervised clustering 'Pam' based approach was applied to identify different molecular isoforms based on the expression of these candidate genes using the "ConsensusClusterPlus" [24] package. The Kaplan–Meier method was applied to compare the overall survival (OS), disease-specific survival (DSS), progression-free interval (PFI), and disease-free interval (DFI) of distinct clusters in the TCGA-LIHC cohort. We further determined whether the expression of these genes differed in different clusters.

#### Construction and validation of BMR

The least absolute shrinkage and selection operator (LASSO) was applied to select genes to construct the BMR risk score with the "glmnet" [25] package.

The risk score was determined by expressing the value of the gene multiplied by the variable coefficient as follows.

$${\text{Risk}}\;{\text{score}} = \left( {{\text{expression}}\;{\text{level}}\;{\text{of}}\;{\text{gene}}_{{\text{A}}} *{\text{coefficient}}\;{\text{of}}\;{\text{gene}}_{{\text{A}}} } \right) + \left( {{\text{expression}}\;{\text{level}}\;{\text{of}}\;{\text{gene}}_{{\text{B}}} *{\text{coefficient}}\;{\text{of}}\;{\text{gene}}_{{\text{B}}} } \right) + \left( {{\text{expression}}\;{\text{level}}\;{\text{of}}\;{\text{gene}}_{{\text{i}}} *{\text{coefficient}}\;{\text{of}}\;{\text{gene}}_{{\text{i}}} } \right)$$

The median risk score was used to stratify patients into low- and high-risk groups. Next, the receiver operating curve (ROC), temporal ROC curve, Kaplan–Meier (KM) method, principal component analysis (PCA) and t-Distributed Stochastic Neighbor Embedding (t-NSE) were applied to assess the performance of BMR in predicting prognosis in patients with HCC by diverse packages. Furthermore, the risk scores and other clinical indicators were analyzed by univariate and multivariate Cox regression analysis using the "survival" package to determine whether BMR acted as an independent risk factor for prognosis. In addition, risk scores for all patients in the ICGC cohort were generated using the same formula as in the TCGA cohort, and the predictive performance of BMR for prognosis was verified in the ICGC cohort.

#### Comparison of BMR with other studies

To determine whether BMR conferred a clinical benefit for predicting OS in HCC patients, we conducted additional analyses, including Decision Curve Analysis (DCA) and Concordance Index analysis. These assessments were performed using the "ggDCA", "rms" [26], and "survival" packages to compare BMR with other relevant studies.

### Somatic mutation analysis

Since gene mutations may impact the prognosis of patients with HCC, we further determined whether somatic mutations differed between those subgroups. Accordingly, we analyzed the top 20 mutated genes in HCC patients in the two subgroups, and waterfall plots were generated to visualize these 20 genes by the "maftool" [27] package. Additionally, we calculated the tumor mutation burden (TMB) for each patient with HCC and examined whether there were any significant differences in TMB between these subgroups. Notably, the impact of TMB on HCC prognosis was explored using the "survival" package.

### Molecular function analysis

We gathered the genes involved in tumorigenesis and progression pathways and used the ssGSEA algorithm to compute the enrichment scores for each pathway in every sample. We further identified whether the scores for these pathways varied between these subgroups. To investigate the molecular mechanisms underlying the difference in prognosis between patients in the high-risk and low-risk groups, we filtered the  DEGs (Padj < 0.05 and |logFC)|≥ 1) between both groups using the "limma" package [20]. The function of DEGs was evaluated by Gene Ontology (GO) and Kyoto Encyclopedia of Genes and Genomes (KEGG) using the "enrichplot", "circlize" and "clusterProfiler" [28] packages.

### The role of BMR in immune features

The tumor microenvironment (TME) comprises essential components such as immune cells and BM, which may interact and influence each other. The "estimate" [29] package was applied to assess the proportion of immune-matrix components in TME, which consisted of the following three main scores: Immune score, Stromal score, and ESTIMATE score. Moreover, we quantified immune cells and immune function using single-sample sequence set enrichment analysis (ssGSEA) and the CIBERSORT algorithm and then further applied the "ggpubr" package to explore whether immune cell infiltration and immune function differed between the two subgroups. In addition, we examined the correlation between the expression of immune checkpoints and risk scores.

It has been reported that potential immune checkpoint blockade (ICB) responses in patients could be predicted by the "TIDE" algorithm (http://tide.dfci.harvard.edu/login/) [30]. Using the TIDE algorithm, we determined the TIDE scores for each sample and examined if there were any differences in TIDE scores between the subgroups. Additionally, we utilized the "TCIA" algorithm (https://tcia.at/home) to identify whether patients in both subgroups were responsive to treatment with PD-1 and CTLA-4. Recent studies have shown that T-cell inflammation score (TIS), FNAP signature, CD8A, and STAT1 are reliable indicators for assessing the effectiveness of immunotherapy [31–33]. Therefore, we investigated whether there were any differences in TIS, FNAP, CD8A, and STAT1 between the subgroups.

### Stemness indices analysis

The mRNA expression-based stemness index (mRNAsi) can reportedly be calculated by the one-class logistic regression (OCLR) algorithm [34]. In this study, we applied the OCLR algorithm to  compute the mRNAsi for each sample and identified differences in mRNAsi between the two subgroups.

### The role of BMR in assessing the response to TACE therapy

In the GSE104580 cohort, we computed the risk scores for each sample and verified whether risk scores differed between responders and non-responders. Furthermore, we explored the AUC values for evaluating the efficacy of TACE by risk score.

### Drug sensitivity

The "pRRophetic" [35] package was used to select potentially effective drugs from more than 300 drugs for both subgroups of patients. We performed a correlation analysis between drug sensitivity and risk scores, using IC50 values as a sensitivity indicator for this investigation.

### Statistical analyses

OS, DSS, and PFI were compared between different subgroups using the Kaplan–Meier method and the log-rank test. We applied the "ComplexHeatmap" [36] package to explore the correlation between risk scores and clinical indicators. Then, we developed and validated the nomogram with the "rms" [26] and "regplot" packages. In our study, statistical analyses were performed by R 4.1.3 and Perl software. *P* value < 0.05 was statistically significant.

## Results

### The BMR signature exhibited excellent prognostic performance 

#### Identification of Basement membrane-related subtypes in HCC

**Fig. 1:**
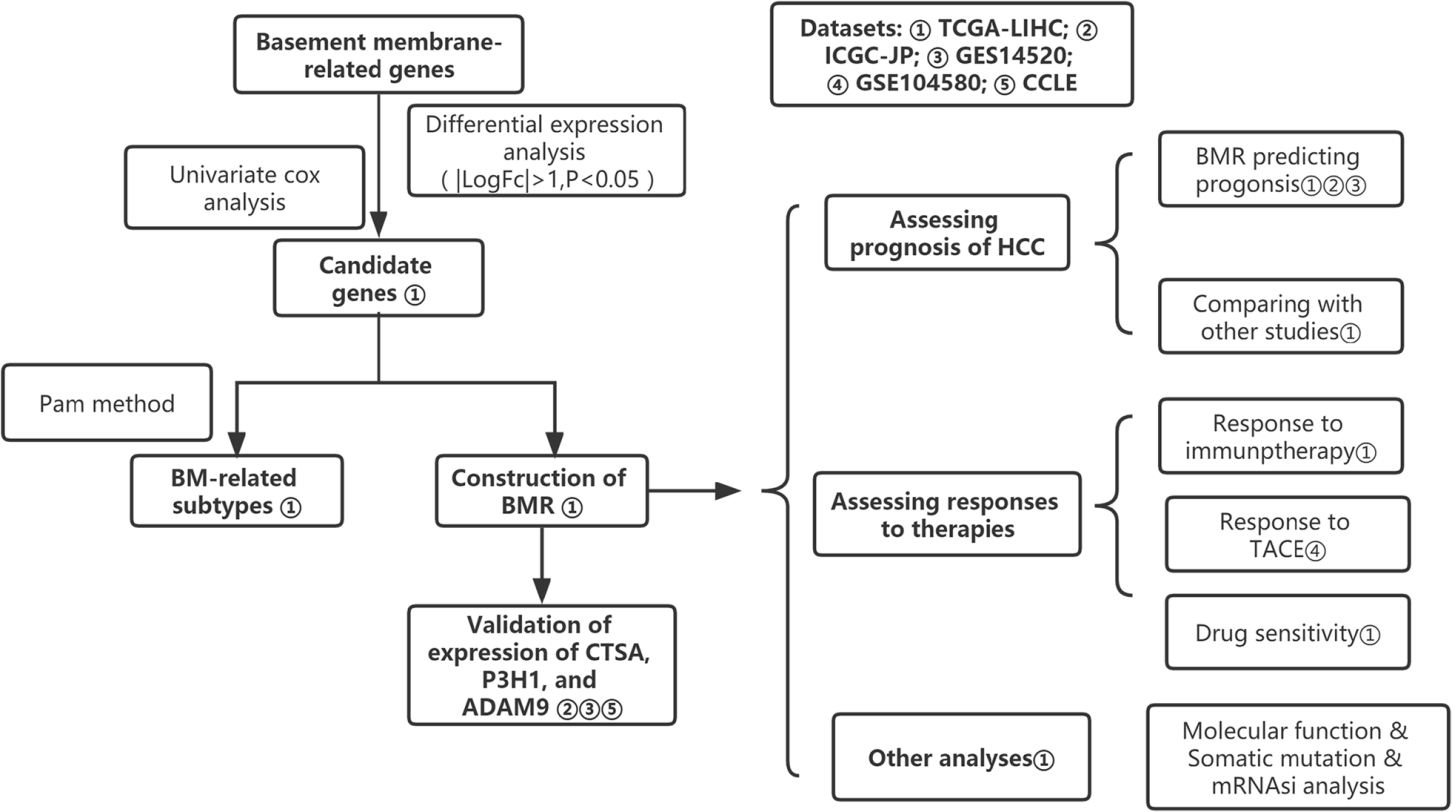
Flowchart of overall study design

**Fig. 2:**
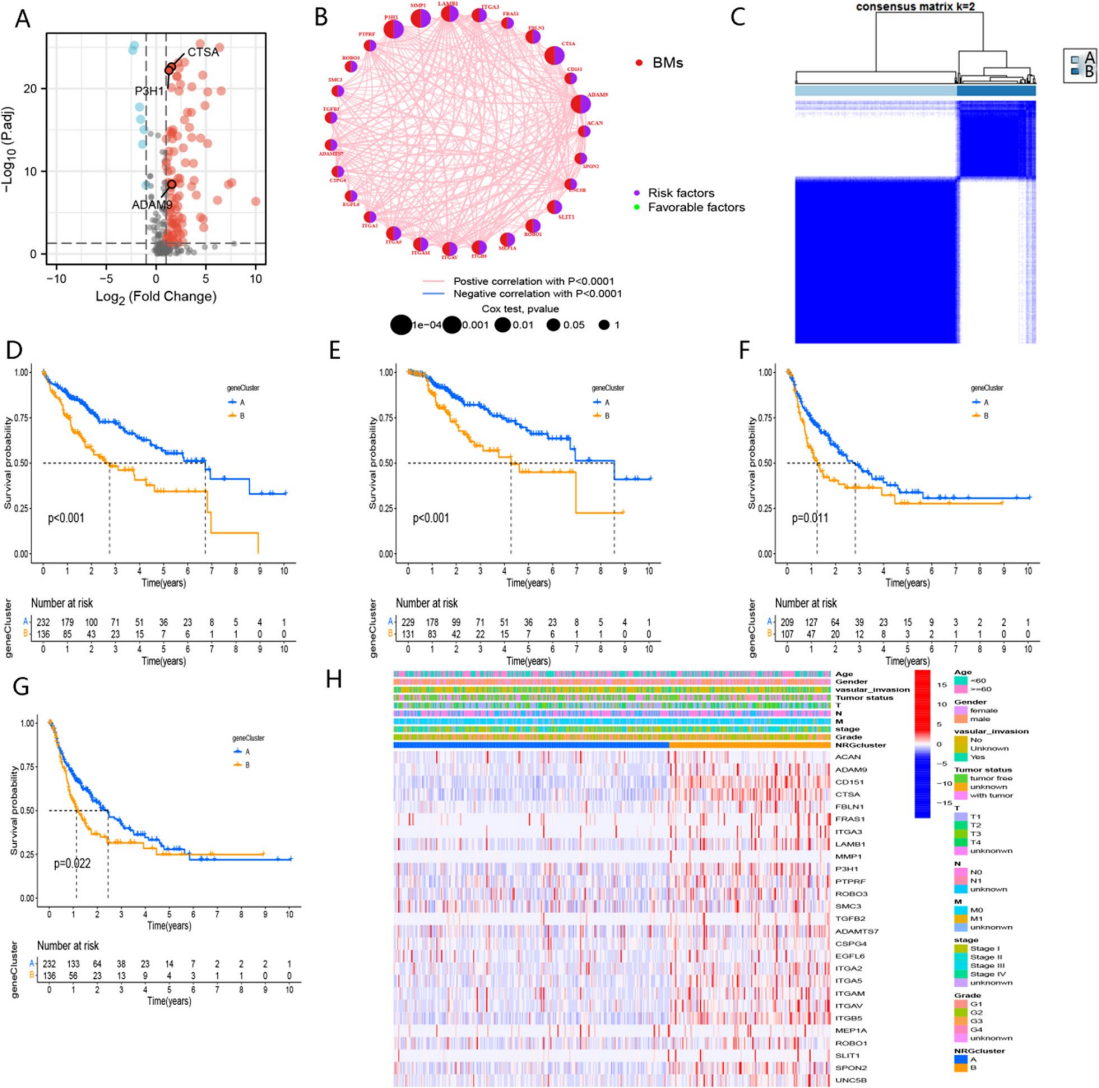
Data processing before the construction of BMR signature **A** Differential expression of basement membrane-related genes in HCC tissues versus normal tissues in the TCGA-LIHC cohort. **B** The correlation network of candidate genes in the TCGA-LIHC cohort. **C** Unsupervised classification of candidate genes in the TCGA-LIHC cohort. **D** Comparison of OS between A and B clusters in the TCGA-LIHC cohort. **E** Comparison of DSS between A and B clustersin the TCGA-LIHC cohort. **F** Comparison of DFI between A and B clusters in the TCGA-LIHC cohort. **G** Comparison of PFI between A and B clusters in the TCGA-LIHC cohort. **H** Comparison of the expression levels of candidate genes in cluster A and cluster B in the TCGA-LIHC cohort

The flow diagram of the study is illustrated in Fig. 1. First, we identified 109 differentially expressed genes among 222 basement membrane-related genes (Fig. 2A, Additional file 1). We further selected 27 candidate genes among these 109 genes, and the correlation network is shown in Fig. 2B.

Furthermore, we employed an unsupervised clustering approach to classify HCC patients into two distinct clusters, A and B, based on the expression levels of genes that influenced prognosis (Fig. 2C). Notably, the survival rate of patients in cluster B was significantly lower than in cluster A (Fig. 2D–G). Moreover, enhanced expression of these candidate genes was observed in cluster B (Fig. 2H).

#### Construction and validation of BMR

**Fig. 3:**
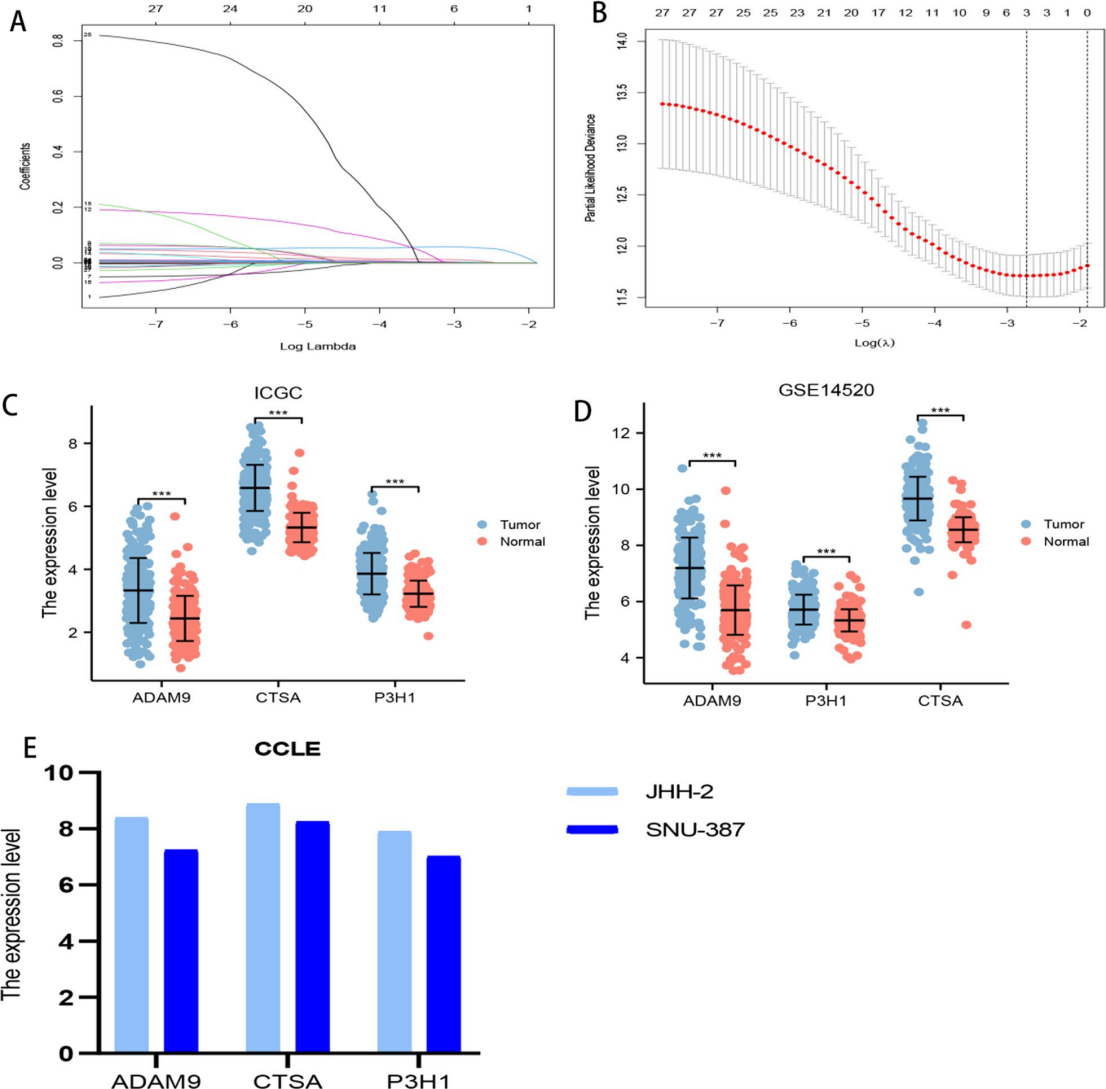
**A** Lasso coefficient profiles. **B** Candidate basement membrane-related genes were filtered by the Lasso algorithm. **C** Comparison of the expression levels of ADAM9, CTSA, and P3H1 between HCC and normal tissues in the ICGC-JP dataset. **D** Comparison of the expression levels of ADAM9, CTSA, and P3H1 between HCC and normal tissues in the GSE14520 dataset. **E** Identification of the expression levels of ADAM9, CTSA, and P3H1 in JHH-2 and SNU-387 cells in the CCLE dataset

The 27 candidate genes identified were subjected to LASSO analysis, which resulted in the selection of 3 genes, namely ADAM9, CTSA, and P3H1, to establish the BMR signature (Fig. 3A, B). Analysis of the ICGC-JP and GSE14520 datasets revealed that these 3 genes were overexpressed in HCC tissues (Fig. 3C, D). Additionally, the expression levels of CTSA, ADAM9, and P3H1 in HCC cell lines were confirmed using the CCLE database (Fig. 3E).

$${\text{BMR}}\;{\text{risk}}\;{\text{score}} = \left[ {{\text{expression}}\;{\text{of}}\;{\text{ADAM}}9 \times \left( {0.008810} \right)} \right] + \left[ {{\text{expression}}\;{\text{of}}\;{\text{CTSA}} \times \left( {0.002283} \right)} \right] + \left[ {{\text{expression}}\;{\text{of}}\;{\text{P}}3{\text{H}}1 \times \left( {0.055080} \right)} \right]$$

**Fig. 4:**
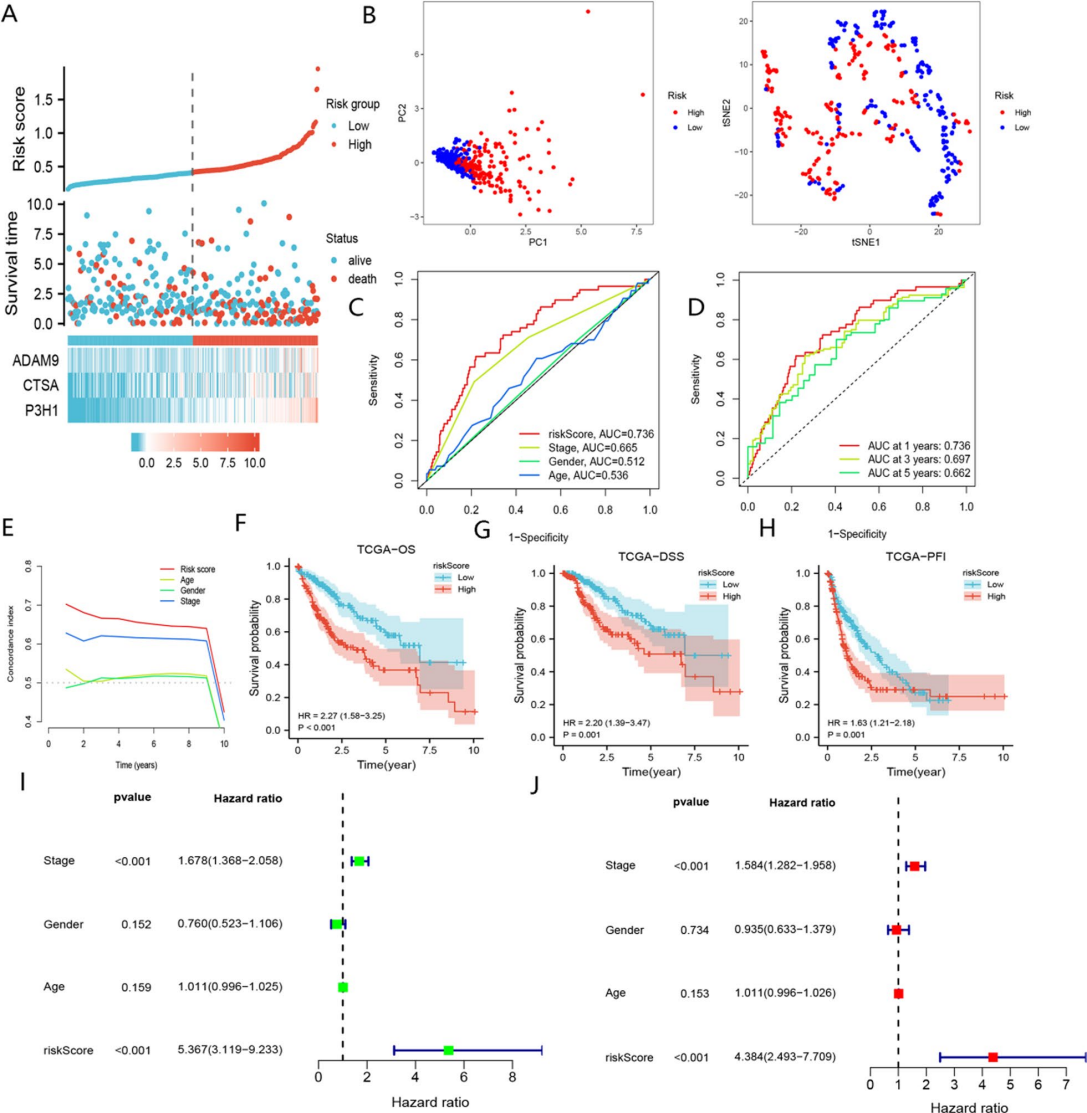
Assessment of the prognostic signature (BMR) in the TCGA-LIHC cohort. **A** Survival status distribution of HCC patients in low- and high-risk groups. **B** PCA analysis of low- and high-risk groups; t-NSE analysis of low- and high-risk groups. **C** ROC curve of age, gender, stage, and risk score. **D** timeROC curve of risk score. **E** C-index curve of age, gender, stage, and risk score. **F** Comparison of OS between low- and high-risk groups. **G** Comparison of DSS between low- and high-risk groups. **H** Comparison of PFI between low- and high-risk groups. **I** Univariate Cox analysis of risk score, gender, stage, and age. **J** Multivariate Cox analysis of risk score and stage

**Fig. 5:**
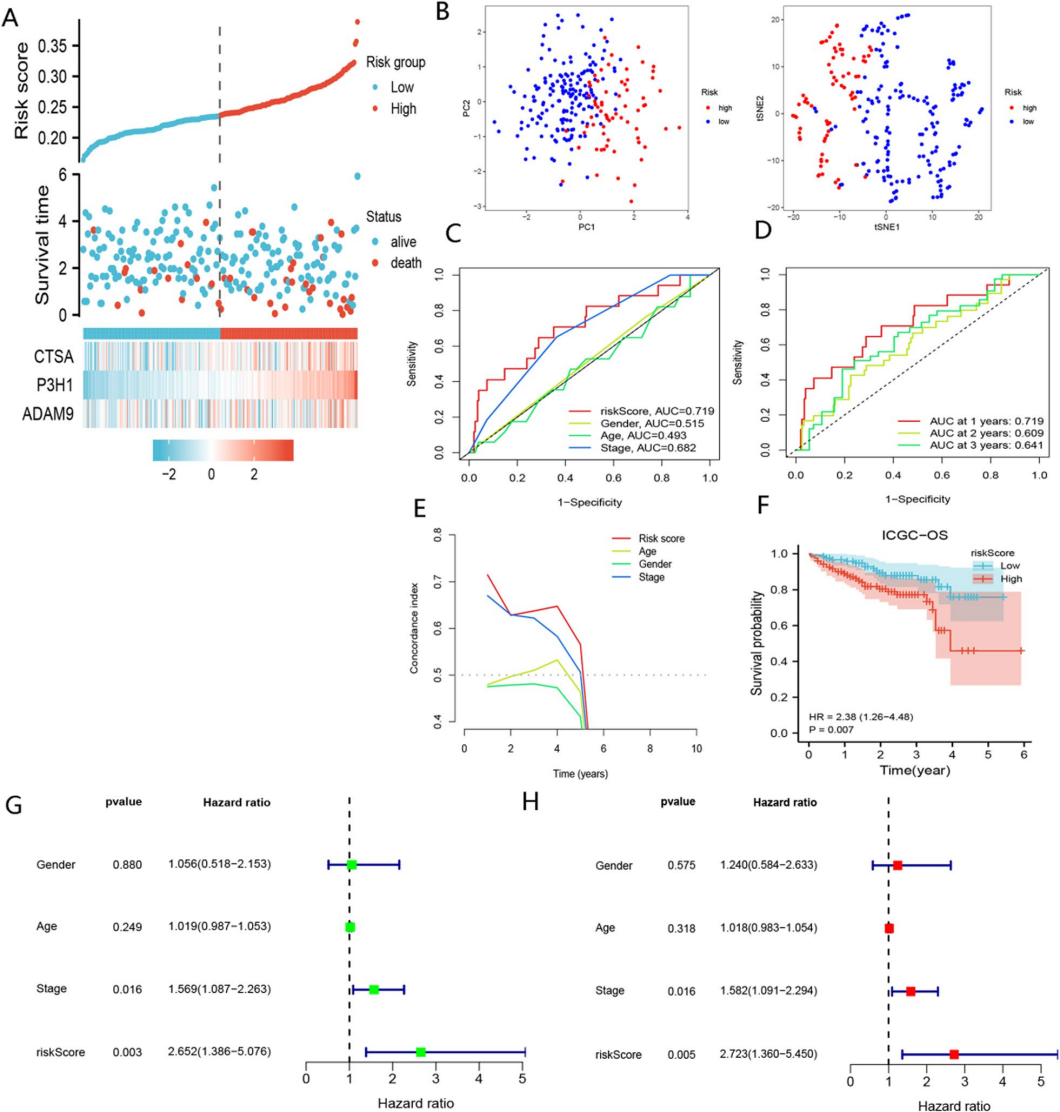
Validation of the prognostic signature (BMR) in the ICGC-JP cohort. **A** Survival status distribution of HCC patients in low- and high-risk groups. **B** PCA analysis of low- and high-risk groups; t-NSE analysis of low- and high-risk groups. **C** ROC curve of age, gender, stage, and risk score. **D** timeROC curve of risk score. **E** C-index curve of age, gender, stage, and risk score. **F** Comparison of OS between low- and high-risk groups. **G** Univariate Cox analysis of risk score, gender, stage, and age. **H** Multivariate Cox analysis of risk score and stage

According to the above formula, the risk scores of HCC patients in the TCGA cohort were obtained, and the HCC patients in the TCGA-LIHC cohort were assigned to the high- and low-risk groups according to the median risk score. Our analysis showed that patients with higher risk scores had a lower OS and increased mortality (Fig. 4A). PCA and t-NSE analyses revealed a significant clustering of HCC patients into low- and high-risk groups (Fig. 4B). As shown in Fig. 4C, D, BMR was a strong predictor of OS in patients with HCC in the TCGA-LIHC cohort. KM curves demonstrated worse prognoses for patients in the high-risk group (*P* < 0.01) (Fig. 4F–H). Our findings indicated that BMR outperformed TNM stage, age, and gender in predicting the prognosis of HCC patients (Fig. 4E). Furthermore, we found that BMR represented an independent risk factor for OS in HCC patients (Fig. 4I, J). The median risk score was used to classify patients in the ICGC-JP cohort into low- and high-risk groups. Fig. 5A–H demonstrated that the results of the ICGC-JP cohort were comparable to those of the TCGA-LIHC cohort. Overall, our BMR signature could be a valuable tool for predicting the prognosis of HCC patients (The baseline clinical characteristics of patients in the TCGA-LIHC cohort and ICGC-JP cohort are provided in  Additional file 2).

Additional file 3 demonstrated that in the  GSE14520 cohort, the risk score was an independent risk factor for unfavorable prognoses in HCC. Notably, TNM-stage, CLIP-satge, and BCLC-stage were not identified as independent risk factors for poor prognosis of HCC, which further highlights the remarkable predictive performance of the BMR signature for HCC prognoses.

#### Correlation of the BMR risk score with clinical parameters

**Fig. 6:**
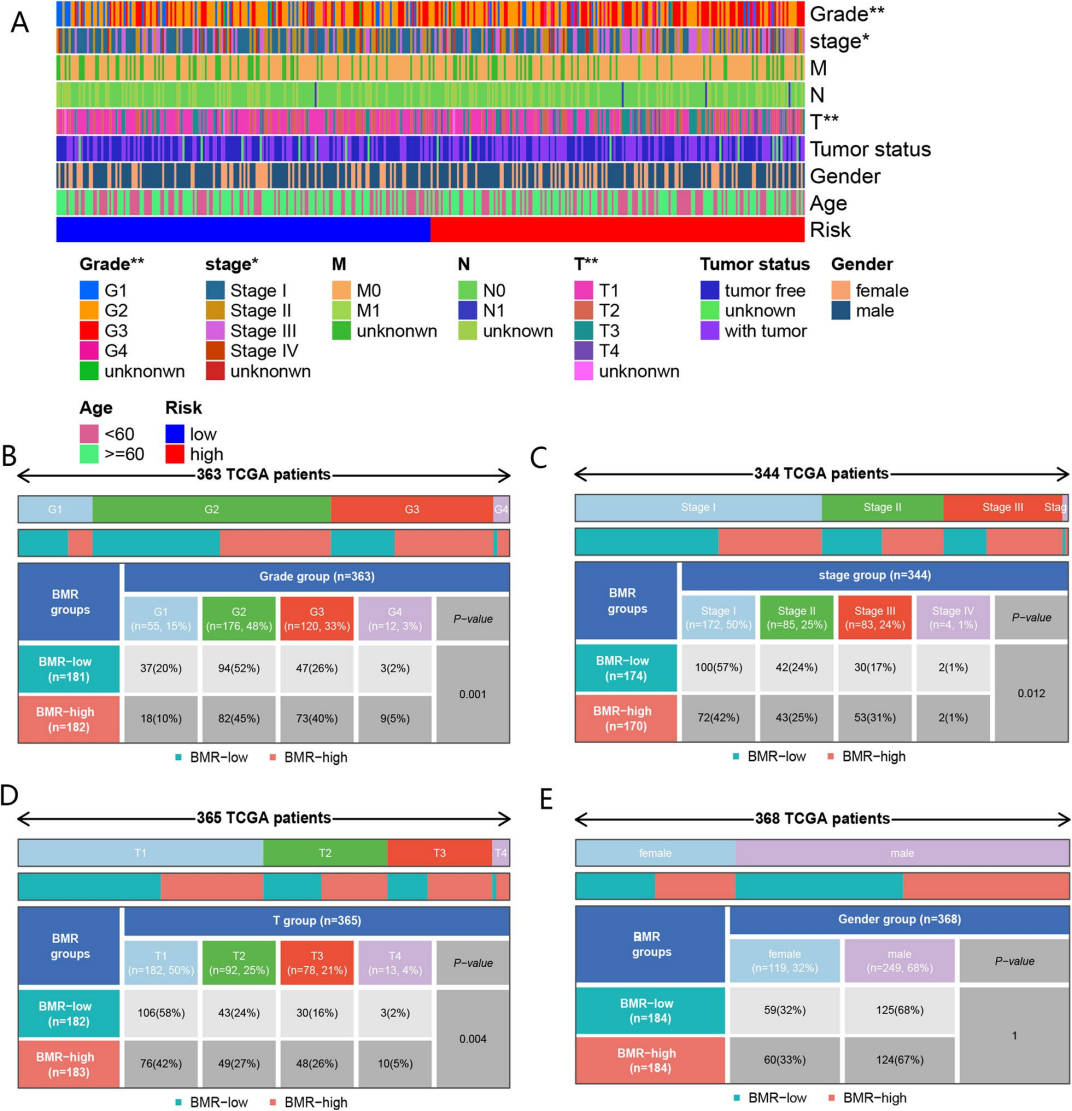
The correlation between BMR and clinical indicators. **A** The correlation heatmap about BMR and common clinical indicators. **B** Comparison of tumor grade between the low- and high-risk groups. **C** Comparison of tumor stage between low- and high-risk groups. **D** Comparison of T-stage between low- and high-risk groups. **E** Comparison of gender between low- and high-risk groups. (* and ** representing *p* < 0.05 and *p* < 0.01,respectively.)

Patients with advanced pathological stage and tumor grade had higher risk scores (Fig. 6A–D), which further validated the prognostic significance of BMR in HCC. These results suggest a higher risk score may indicate a poorer prognosis in HCC patients. However, there were no significant associations between risk scores and sex, age group, or tumor status (Fig. 6A, E).

#### Comparison of BMR with other gene signatures

**Fig. 7:**
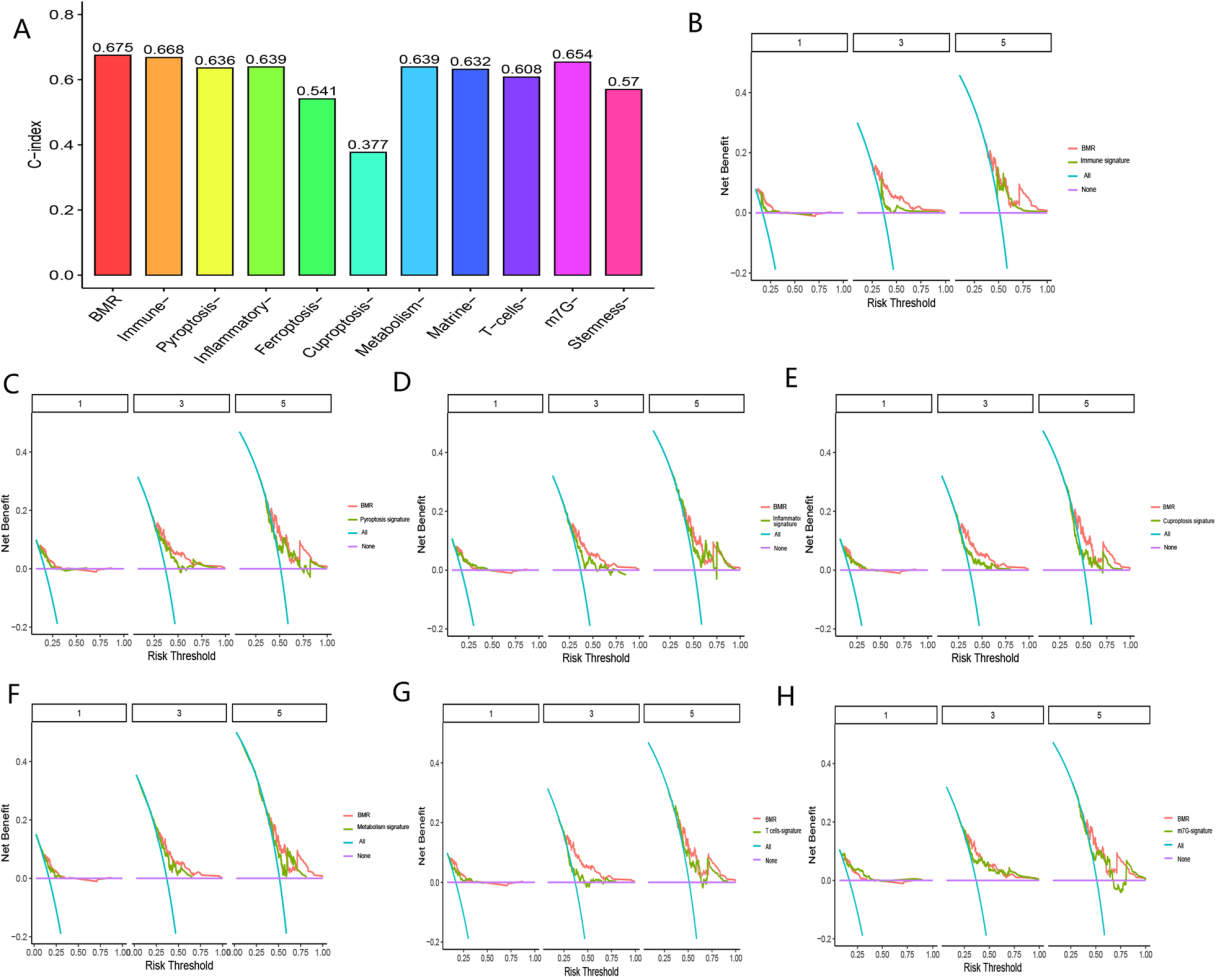
**A** C-index curve of BMR and other studies. **B**–**H** Decision curve analysis of BMR with other gene signatures

Our findings revealed that the C-index of BMR was 0.675, which was higher than the C-index values reported in other studies (Fig. 7A). When compared to the cuproptosis-, immune-, pyroptosis-, inflammatory response-, ferroptosis-, and metabolism-related signatures reported in previous studies [37–46], BMR demonstrated greater clinical applicability in evaluating the prognoses of HCC (Fig. 7B–H). These results further validated the strong predictive ability of BMR in assessing the OS of HCC patients.

#### Construction of a BMR-based nomogram

**Fig. 8:**
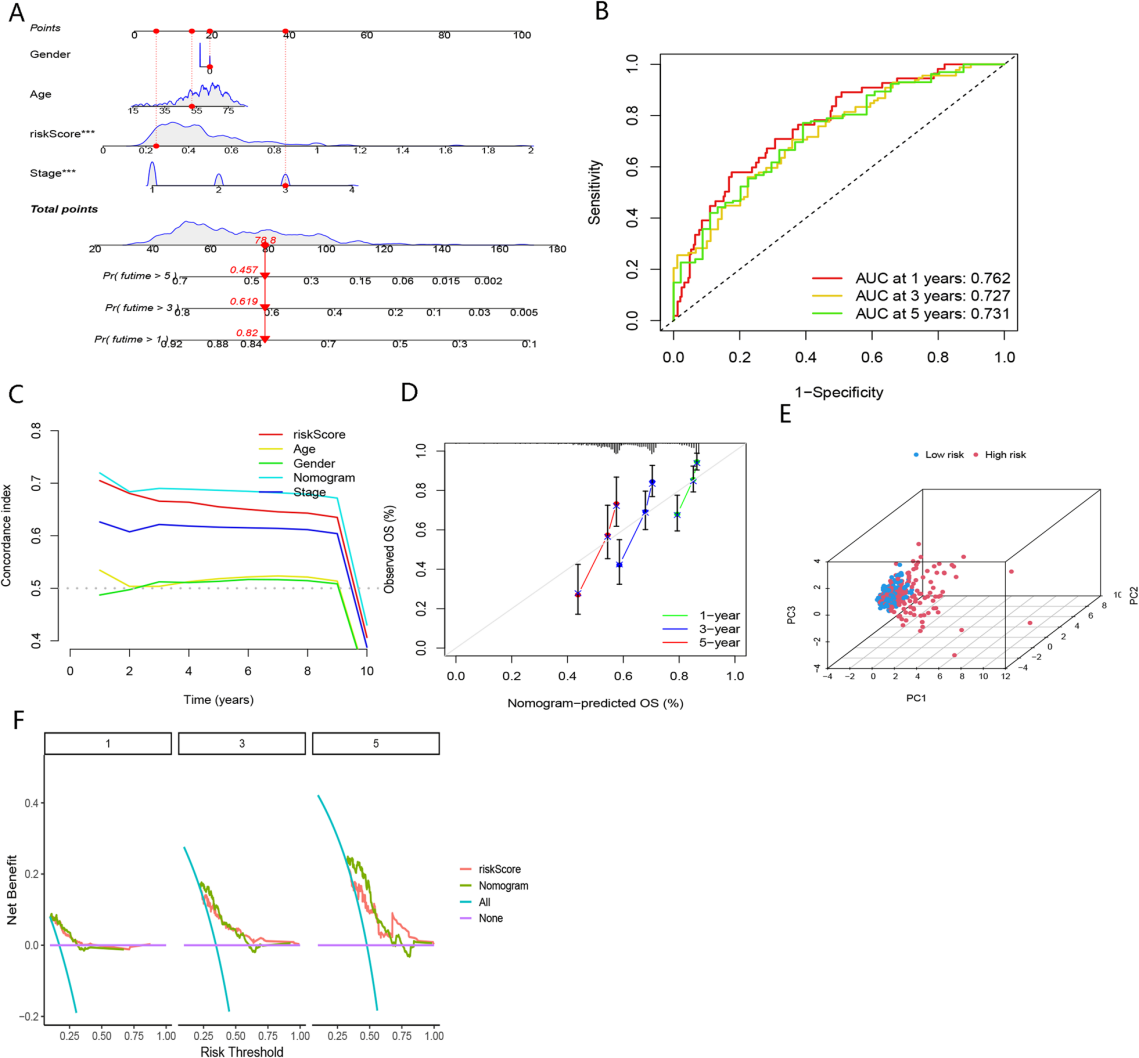
Nomogram based on BMR, stage, gender, and stage for prediction of OS in TCGA-LIHC cohort. **A** Nomgram based on BMR, stage, gender, and stage in TCGA-LIHC cohort. **B** timeROC curve of the nomogram. **C** C-index curve of the nomogram, age, gender, stage, and risk score. **D** Calibration curve of the nomogram. **E** PCA plot of the nomogram. **F** DCA curve of BMR and nomogram

We created a nomogram in the TCGA-LIHC cohort based on the risk score and other clinical indicators for the prediction of OS (Fig. 8A). As illustrated in Fig. 8B, C, F, the nomogram exhibited superior performance in accurately predicting prognoses of HCC than other features. The calibration curves indicated that the predicted survival values for 1-year, 3-year, and 5-year were highly consistent with the actual values (Fig. 8D). Based on the total score obtained from the nomogram, we observed a significant clustering of HCC patients (Fig. 8E). These results suggest that our nomogram based on risk score has huge prospects for accurately predicting the OS of HCC patients.

### The role of BMR in mRNAsi analysis

**Fig. 9:**
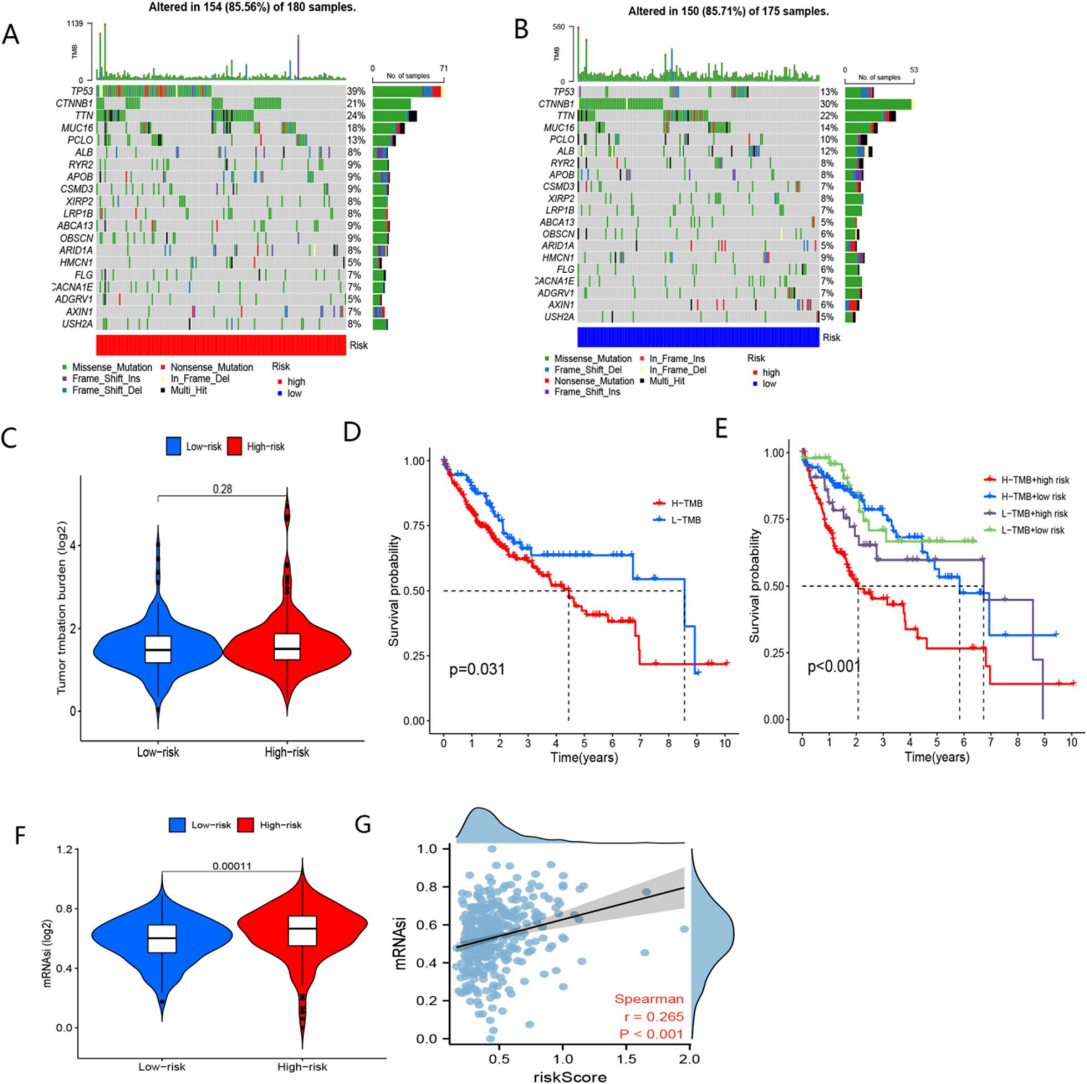
Somatic mutation and mRNAsi analyses in TCGA-LIHC cohort. **A** Matfools of the high-risk group. **B** Matfools of the low-risk group. **C** Comparison of TMB between high- and low-risk groups. **D** Comparsion of OS between the low-TMB and high-TMB groups. **E** Comparsion of OS between low-TMB + low-risk, low-TMB + high-risk, high-TMB + low-risk, and high-TMB + high-risk groups. **F** Comparison of mRNAsi between high- and low-risk groups. **G** The correlation between risk score and mRNAsi

The high-risk group showed an increase in mRNAsi and a positive correlation between the risk score and mRNAsi (Fig. 9A, B). The results imply that the poorer prognosis observed in the high-risk group may be attributed to mechanisms associated with mRNAsi.

### The role of BMR in assessing the somatic mutation

Waterfall plots were generated to visualize the top 20 genes mutated in these subgroups (Fig. 9C, D). Our results indicated that TP53 mutations were more common in the high-risk group, while CTNNB1 mutations were more prevalent in the other group. As shown in Fig. 9E, TMB was not significantly different between these subgroups. The OS of patients in the high-TMB group was significantly lower than in the low-TMB group (Fig. 9F). Interestingly, the OS of patients in the high-TMB group was significantly lower than in the low-TMB group (Fig. 9F). It is worth noting that the combination of TMB with risk score demonstrated higher OS in the group with low risk and high TMB (*P* < 0.001) (Fig. 9G).

### The role of BMR in molecular function analysis

**Fig. 10:**
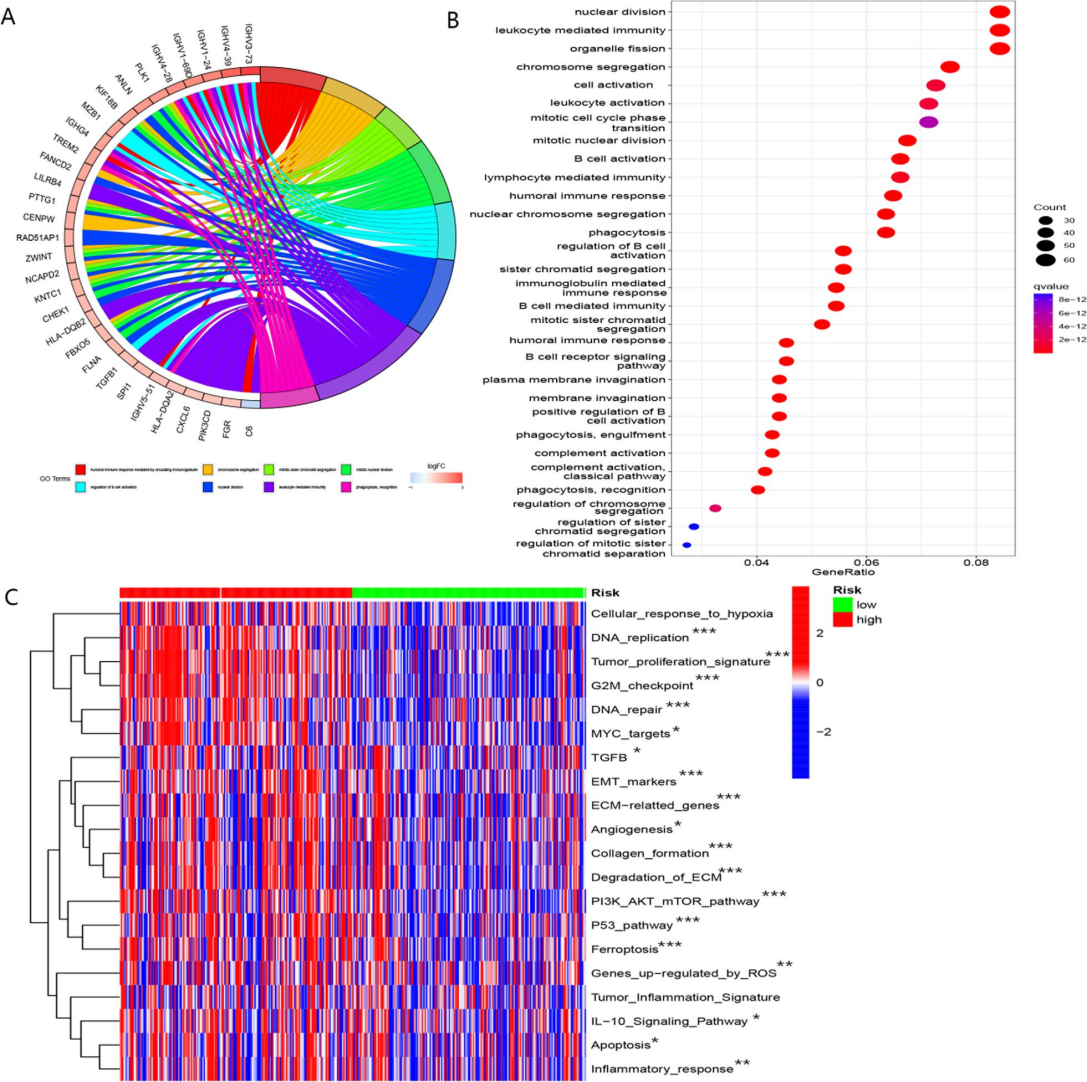
Functional analysis of DEGs between high- and low-risk groups based on TCGA-LIHC cohort. **A** Go analysis of DEGs between high- and low-risk groups. **B** KEGG analysis of DEGs between high- and low-risk groups. **C** Comparison of tumor-related pathways between low- and high-risk groups

As shown in Fig. 10A, GO analyses revealed that the DEGs of the high- and low-risk groups were significantl enriched in pathways mediating immune function. KEGG analysis demonstrated that the DEGs were associated with nuclear division and organelle fission (Fig. 10B). Furthermore, apoptosis, DNA repair, ferroptosis, and angiogenesis pathways related to oncogenesis and tumor progression were significantly enriched in patients belonging to the high-risk group (Fig. 10C).

### The role of BMR in immune profiling

#### The correlation between BMR and immune features

**Fig. 11:**
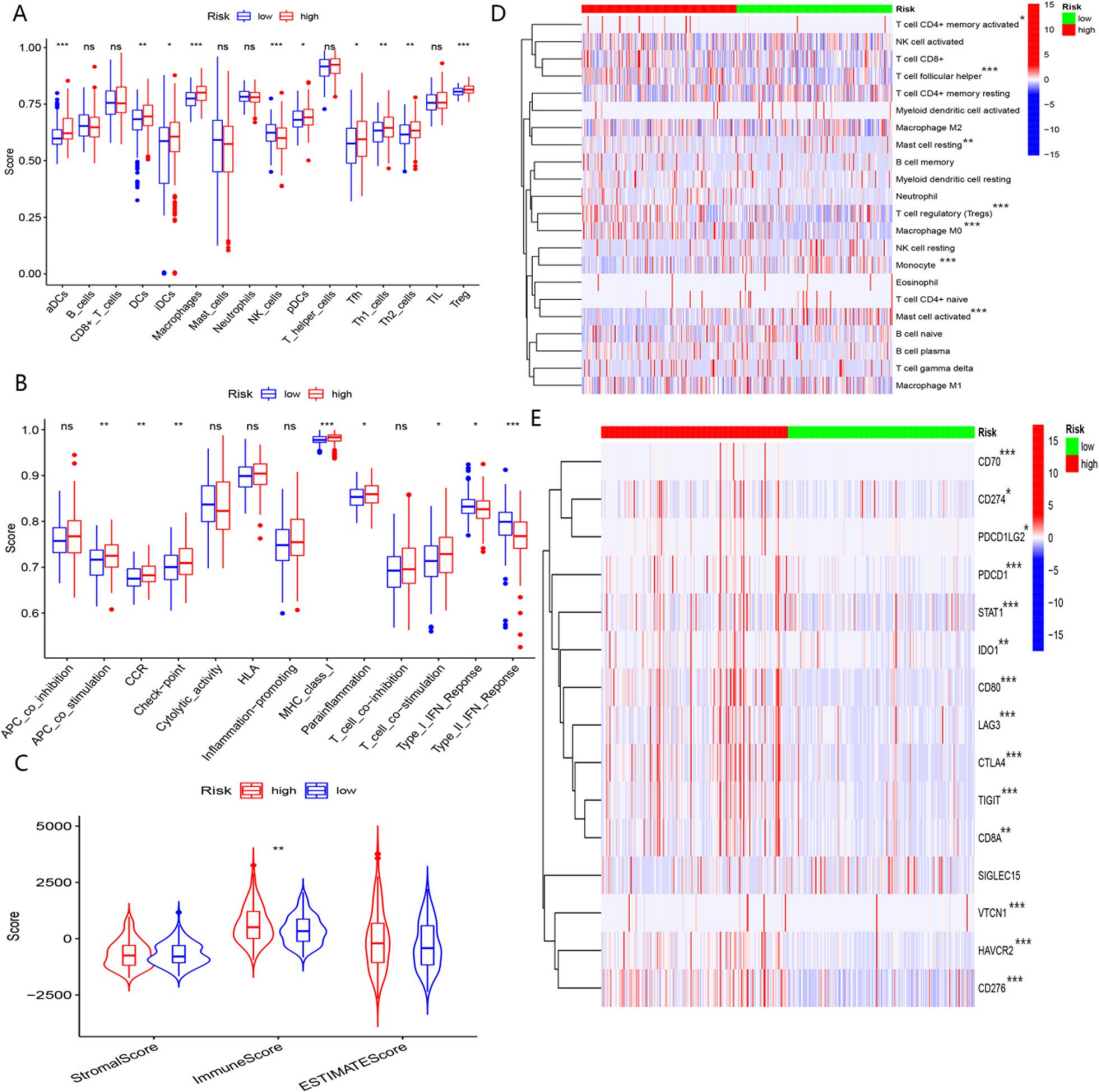
The analyses of immune features in the TCGA-LIHC cohort. **A** Comparison of immune cell infiltration between high- and low-risk groups with ssGSEA algorithm. **B** Comparison of immune function between high- and low-risk groups with ssGSEA algorithm. **C** Comparison of ESTIMATE score, stromal score, and immune score. **D** Comparison of immune cell infiltration between high- and low-risk groups with CIBERSORT algorithm. **E** Comparison of expression levels of immune checkpoints between the high- and low-risk groups. (*, **, *** representing *p* < 0.05, *p* < 0.01 and *p* < 0.001,respectively.)

Our study showed that patients in the high-risk group had increased immune scores (Fig. 11C). The subgroups did not exhibit significant differences in the ESTIMATE or Stromal scores. As shown in Fig. 11A, D, patients in the low-risk group demonstrated a more substantial percentage of macrophages and NK cells, although the other group displayed a higher abundance of Th2 and Treg cells. In terms of immune function, the type-I IFN response and type-II IFN response were significantly enhanced in the low-risk group, while the high-risk group showed higher expression of immune checkpoints (Fig. 11B, E). We found that patients in the high-risk group exhibited immunosuppression.

#### The role of BMR in predicting responses to immunotherapy

Considering the clinical potential of immune checkpoints for immunotherapy, we explored potential distinctions in immune checkpoint expression between the two subgroups (Fig. 11E).

**Fig 12:**
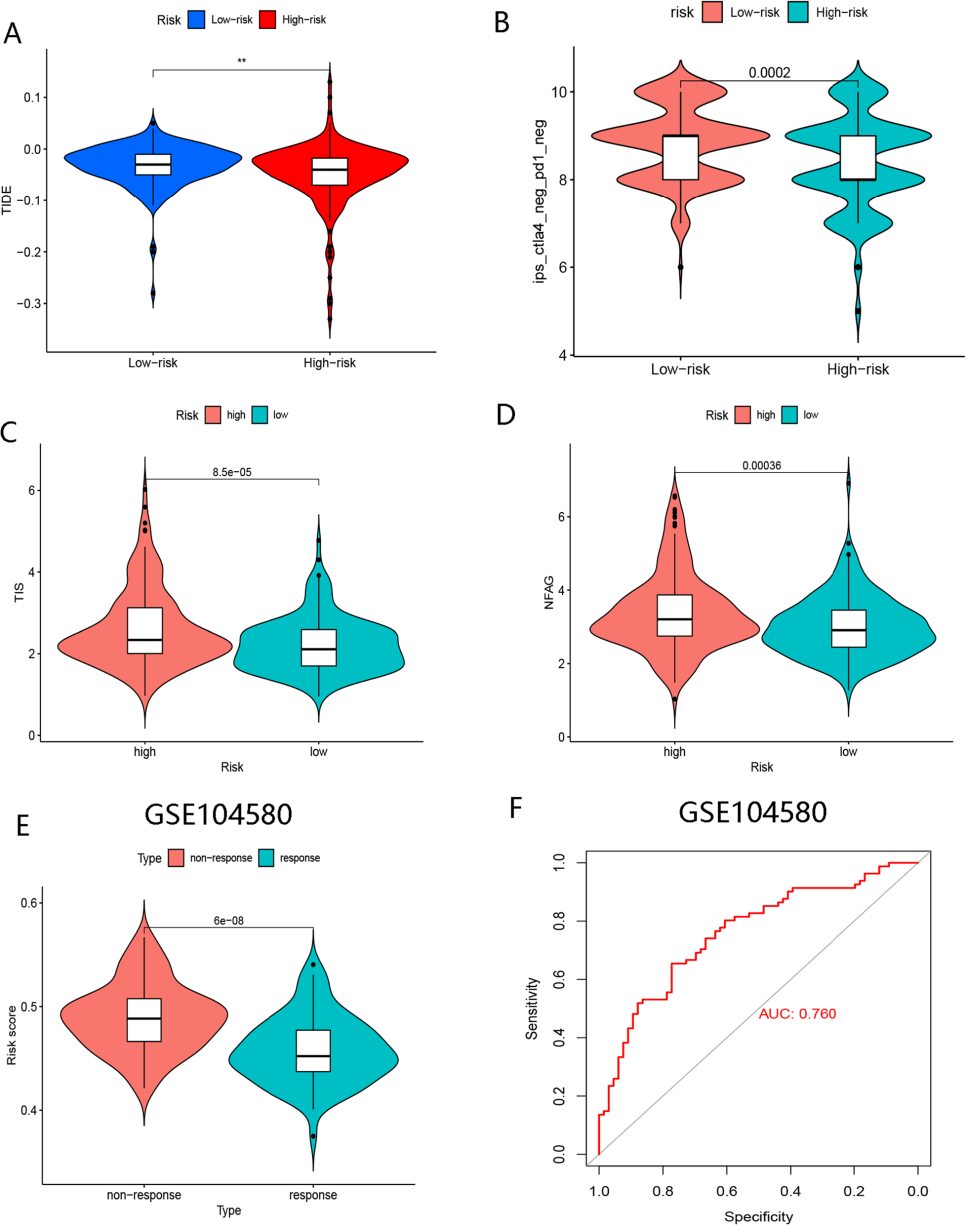
The analysis of response to immunotherapy and TACE. **A** Comparison of TIDE score between high- and low-risk groups. **B** Comparison of IPS score (negative PD-1 and negative CTLA-4) between high- and low-risk groups. **C** Comparison of TIS between the low- and high- risk groups. **D** Comparison of NFAG between low- and high-risk groups. **E** Comparison of risk scores between responders and non-responders to TACE therapy group. (*, **, *** representing *p* < 0.05, *p* < 0.01 and *p* < 0.001,respectively.)

The high-risk group exhibited immunosuppression, as indicated by the lower TIDE score (Fig. 12A). Conversely, it is highly unlikely that the low-risk group would benefit from therapies with PD-1 and CTLA-4, as evidenced by an analysis of the TCIA database (Fig. 12B). Furthermore, the high-risk group showed elevated NFAG and TIS (Fig. 12C, D), suggesting that immunotherapy may improve prognoses for these patients.

### BMR for prediction of responses to TACE therapy

As shown in Fig. 12E, reduced risk scores were observed in TACE-treated responders. The AUC value for the risk score to estimate the efficacy of TACE was 0.760 (Fig. 12F). Accordingly, patients in this subgroup might be more responsive to TACE treatment.

### The role of BMR in predicting drug sensitivity

**Fig 13:**
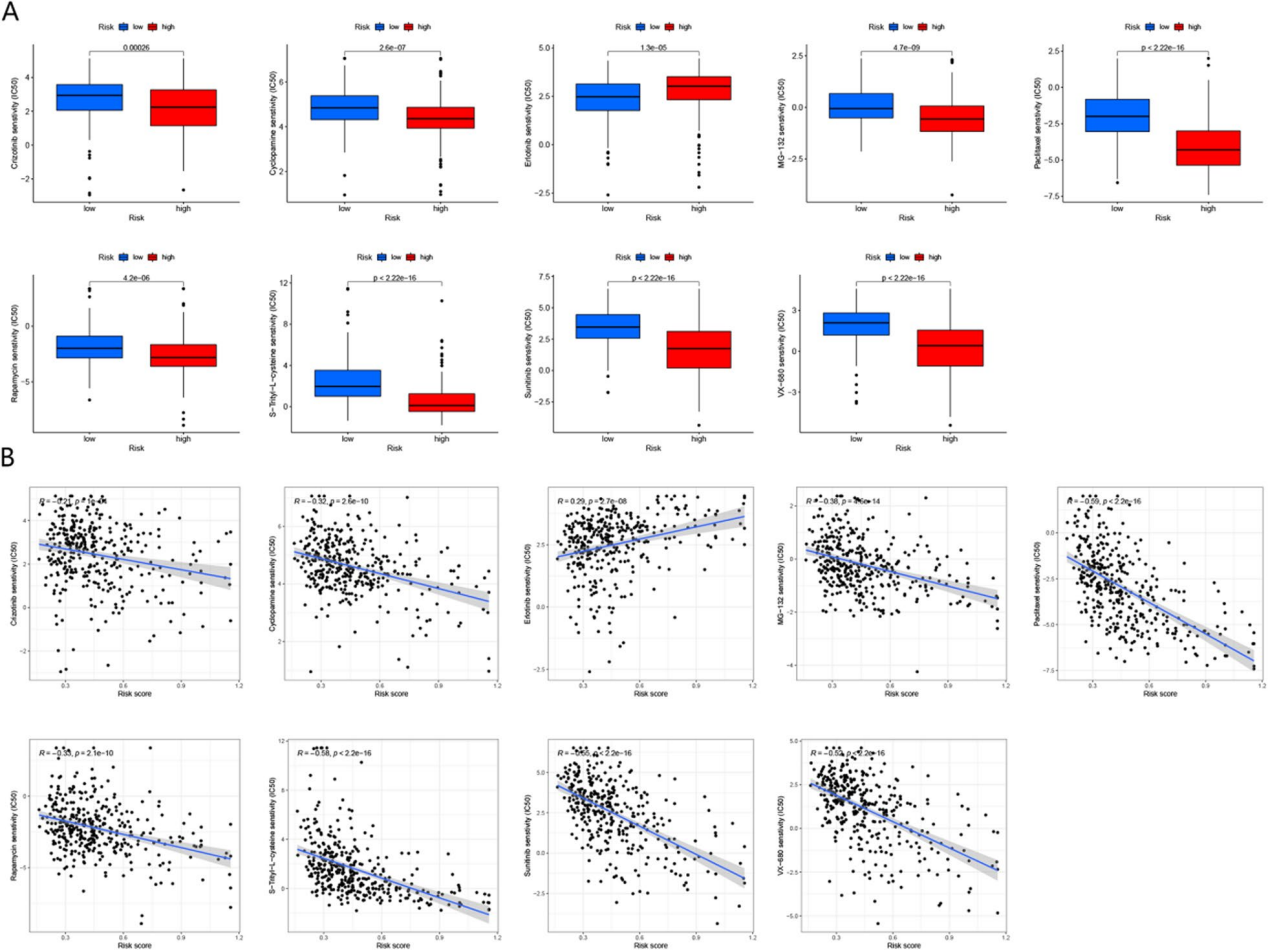
Drug sensitivity analyses based on the TCGA-LIHC cohort. **A** Comparison of drug sensitivity between high- and low-risk groups. **B** The correlation between risk score and drug sensitivity

We identified nine drugs, including "crizotinib", "cyclopamine", "paclitaxel", "MG-132", "rapamycin", "S-Trityl-L-cysteine", "sunitinib" and "VX-680", which appeared more favorable for the high-risk group, whereas "erlotinib" may be more beneficial for another group (Fig. 13A). Furthermore, we computed the correlation coefficient between the risk score and IC50 values (Fig. 13B), indicating that patients belonging to the high-risk group may exhibit a better response to "paclitaxel".

## Discussion

This study utilized an unsupervised clustering approach and demonstrated that BM genes could potentially serve as a prognostic marker for HCC. A BMR signature was established by employing data from the TCGA cohort and then validated in the ICGC cohort and GSE14520 cohort. We found that patients in the high-risk group had the worse prognoses. In addition, we revealed that various pathways related to the development and progression of tumors were significantly enriched in the high-risk group of patients. Overall, we substantiated that patients in the high-risk group might benefit from immunotherapy, whereas patients in the other group might be responsive to TACE treatment. Eventually, effective drugs were screened for patients in these subgroups, respectively. Our results corroborate the importance of BMR in predicting outcomes and guiding therapy for individuals with HCC.

BM has been implicated in promoting invasion, migration and distant metastasis in several types of cancer, including lung cancer, esophageal cancer, breast cancer, and pancreatic adenocarcinoma [47–50]. More importantly, the BM has been proposed as a target for some malignant tumors [51–54]. There is a rich literature available substantiating that the BM is involved in the progression of HCC via multiple mechanisms and that it may be considered as a biomarker for monitoring the prognosis of HCC patients [15, 16]. These findings also suggest that the BM has huge potential as a novel target for HCC therapy. Therefore, studying the role of the BMR in HCC is of great significance. In this study, our BMR signature comprised ADAM9, CTSA and P3H1. It has been established that ADAM9 promotes the progression of advanced HCC and might be applied as a biomarker during immunotherapy for HCC [55, 56]. Cathepsin A (CTSA) is a lysosomal protease established to be a promising biomarker for the diagnosis and prognosis of HCC [57]. Moreover, P3H1 may be associated with the immunosuppressed state of other malignant tumors [58]. However, the role played by P3H1 in HCC is unclear, and our study provided hitherto undocumented evidence that higher expression of P3H1 is positively associated with poor prognosis in HCC.

Data from the ICGC-JP and TCGA-LIHC databases proved that the BMR signature yielded good performance in determining the prognoses of HCC. Notably, patients in the high-risk group exhibited lower survival rates. Additionally, the BMR signature was an independent risk factor for predicting prognosis in HCC. To better implement BMR in clinical practice, we created a nomogram based on clinical features and our risk score. The calibration curves and C-index curves both demonstrated the precise discrimination ability of the nomogram for predicting the prognoses of HCC patients.

Little is currently known about the mechanism underlying the difference in prognoses between the two subgroups, nor is it clear whether a genetic mutation could contribute to this outcome. Accordingly, we performed somatic mutation analysis and found that the most common gene mutation in the high- and low-risk groups were TP53 and CTNNB1, respectively. It has been reported that patients with a higher incidence of CTNNB1 changes in liver cancer presented with smaller tumor sizes and well-differentiated tumors [59]. TP53 mutations are positively correlated with tumor invasion of blood vessels in malignant tumors [60]. As a result, distinct genetic changes between two subgroups may contribute to the  heterogeneity in prognoses. 

In this study, we conducted a functional enrichment analysis of the DEGs. Interestingly, most genes were significantly enriched in immune-related pathways. Thus, we assumed that the mechanism behind the adverse prognosis in the high-risk group might be linked to immune features. Our results revealed that the TME significantly differed between the two subgroups. Regarding immune cells, the high-risk group exhibited an increase in Th2 and Treg cells and a decreased in NK cells. Current evidence suggests that Th2 cells and Treg cells may facilitate the escape of malignant cells from immune system surveillance [61], and NK cells have been demonstrated to be promising immune cells, given their tumor-killing effects [62]. Our study also revealed notable differences in immune function between the two risk groups. In this regard, we observed weaker type-I and type-II IFN reactions in the high-risk group, indicating a significant state of immunosuppression in these patients. Tumor cells in an immunologically suppressed state can escape from immune surveillance, thus contributing to the proliferation of tumors [63].

In addition, our study found that higher mRNAsi was observed in patients of the high-risk group, and enhanced mRNAsi represent more dedifferentiated tumor cells and more aggressive tumors. This finding may help explain the adverse prognoses of high-risk patients from another perspective.

Further investigation is warranted to determine if there are notable differences in the treatment of patients within these subgroups.

Immunotherapy holds great promise as a treatment for many HCC patients. The effectiveness of such therapy depends not only on the ability of immune cells to adequately penetrate the tumor microenvironment but also on the sufficient expression of immune checkpoints within the cancerous tissue. Lately, inhibitors of immune checkpoints (ICIs) aimed at CTLA-4 and PDCD1 have shown promising results in treating patients with HCC [64]. Our findings indicate that high-risk group patients had higher expression levels of common immune checkpoints, suggesting that they may be more responsive to immunotherapy. This was further supported by the TIDE and IPS scores. Additionally, we observed higher TMB in high-risk group patients, and recent studies have shown that higher TMB is associated with better response to immunotherapy. [65]. Notably, low-risk group patients had more CTNNB1 synapses, indicating non-inflammatory T-cell tumor tissues resistant to immunotherapy [66]. Therefore, BMR can provide valuable guidance for immune intervention in HCC patients from various perspectives.

TACE represents a potential therapeutic option for advanced patients with HCC. Our results indicated that low-risk patients might be more sensitive to TACE therapy, and BMR played an active role in determining the effectiveness of TACE therapy for HCC patients. Chemotherapy remains the mainstream treatment modality for many advanced HCC patients. However, with over 300 chemotherapeutic agents, it may be challenging for clinicians to select suitable drugs for patients with HCC. Based on BMR, we screened sensitive chemotherapeutic agents for these subgroups, which may guide physicians to carry out personalized treatment of HCC patients. Our results indicated that high-risk patients might be most susceptible to "paclitaxel".

Few studies have investigated the role of basement membrane-associated gene sets in HCC, and our findings lay the groundwork for further investigations, which hold significant importance. Interestingly, BMR performed better in assessing the prognosis of HCC patients compared to other genetic biomarkers. Indeed, our study was also subject to limitations. Similar to the literature, we utilized public datasets and did not validate these findings on clinical samples. Moreover, the nomogram in our study lacked methemoglobin, which may slightly impact the accuracy of our nomogram in predicting the prognoses of HCC patients.

## Conclusion

To summarize, our study introduced a new gene signature (BMR) consisting of three basement membrane-related genes, which yielded strong predictive performance for the prognosis and response to immunologic and TACE therapies in HCC patients. Moreover, we used BMR to identify potentially effective drugs from a pool of over 300 agents for patients in high- and low-risk groups. Overall, BMR has huge prospects for application as a biomarker for making diagnostic and therapeutic decisions.

## Supplementary Information

**Additional file 1.** A list of 109 differentially expressed genes among 222 basement membrane-related genes between HCC tissues and normal liver tissues in the TCGA-LIHC cohort.

**Additional file 2.** Baseline Characteristics in TCGA-LIHC cohort and ICGC-JP cohort.

**Additional file 3.** Univariate and Multivariate Cox analysis of risk score, gender, age, TNM- stage, CLIP-stage, and BCLC- stage in GSE14520 cohort.

## Data Availability

The data used to support the findings of this study are available from public databases, including TCGA database (https://portal.gdc.cancer.gov/projects/TCGA-LIHC), ICGC database (https://dcc.icgc.org/projects/LIRI-JP), CCLE database (https://portals.broadinstitute.org/ccle), GEO (GSE104580 and GSE14520) database (https://www.ncbi.nlm.nih.gov/geo/).

## Abbreviations

HCC: Hepatocellular carcinoma
LASSO: Least absolute shrinkage and selection operator
TCGA: Cancer Genome Atlas
TMB: Tumor mutation burden
OS: Overall survival
TME: Tumor microenvironment
BMR: Basement-membrane-related regulators
BM: Basement membrane
