# Supplementary material for: Basement membrane-related regulators for prediction of prognoses and responses to diverse therapies in hepatocellular carcinoma
Source: BMC Med Genomics. 2023 Apr 20;16:81. doi: 10.1186/s12920-023-01504-z (PMC10116671; doi:10.1186/s12920-023-01504-z)
Supplement: Supplementary file 2 — Additional file 2. Baseline Characteristics in TCGA-LIHC cohort and ICGC-JP cohort. [file 12920_2023_1504_MOESM2_ESM.docx]

| Characteristics | TCGA-LIHC cohort  (368 patients) | ICGC-JP cohort  (232 patients) |
| --- | --- | --- |
| Age (mean, range) | 59（16,90） | 67 （31,89） |
| Gender (100%) |  |  |
| Female | 119 (32.3%) | 61(26.3%) |
| Male | 249(67.7%) | 171(73.7%) |
| Stage (100%) |  |  |
| Stage I | 172(46.7%) | 36(15.5%) |
| Stage II | 85(23.1%) | 106(45.7%) |
| Stage III | 83(22.6%) | 71(30.6%) |
| Stage IV | 4(1.1%) | 19(80.2%) |
| Unknown | 24(6.5%) | 0 |
| Survival time (Year)  (mean, range) | 2.26 (0.0027-10.07) | 2.22 (0.27-5.92) |
| Survival status (100%) |  |  |
| Alive | 238(64.7%) | 42(18.1%) |
| Death | 130(35.3%) | 190(81.9%) |

Baseline Characteristics in TCGA-LIHC cohort and ICGC-JP cohort
