# Supplementary material for: Basement membrane-related regulators for prediction of prognoses and responses to diverse therapies in hepatocellular carcinoma
Source: BMC Med Genomics. 2023 Apr 20;16:81. doi: 10.1186/s12920-023-01504-z (PMC10116671; doi:10.1186/s12920-023-01504-z)

**Supplementary file 3:**

1. Univariate Cox analysis of risk score, gender, age, TNM- stage, CLIP-stage, and BCLC- stage in GSE14520 cohort. (B) Multivariate Cox analysis of risk score, TNM- stage, CLIP-stage, and BCLC- stage in GSE14520 cohort.


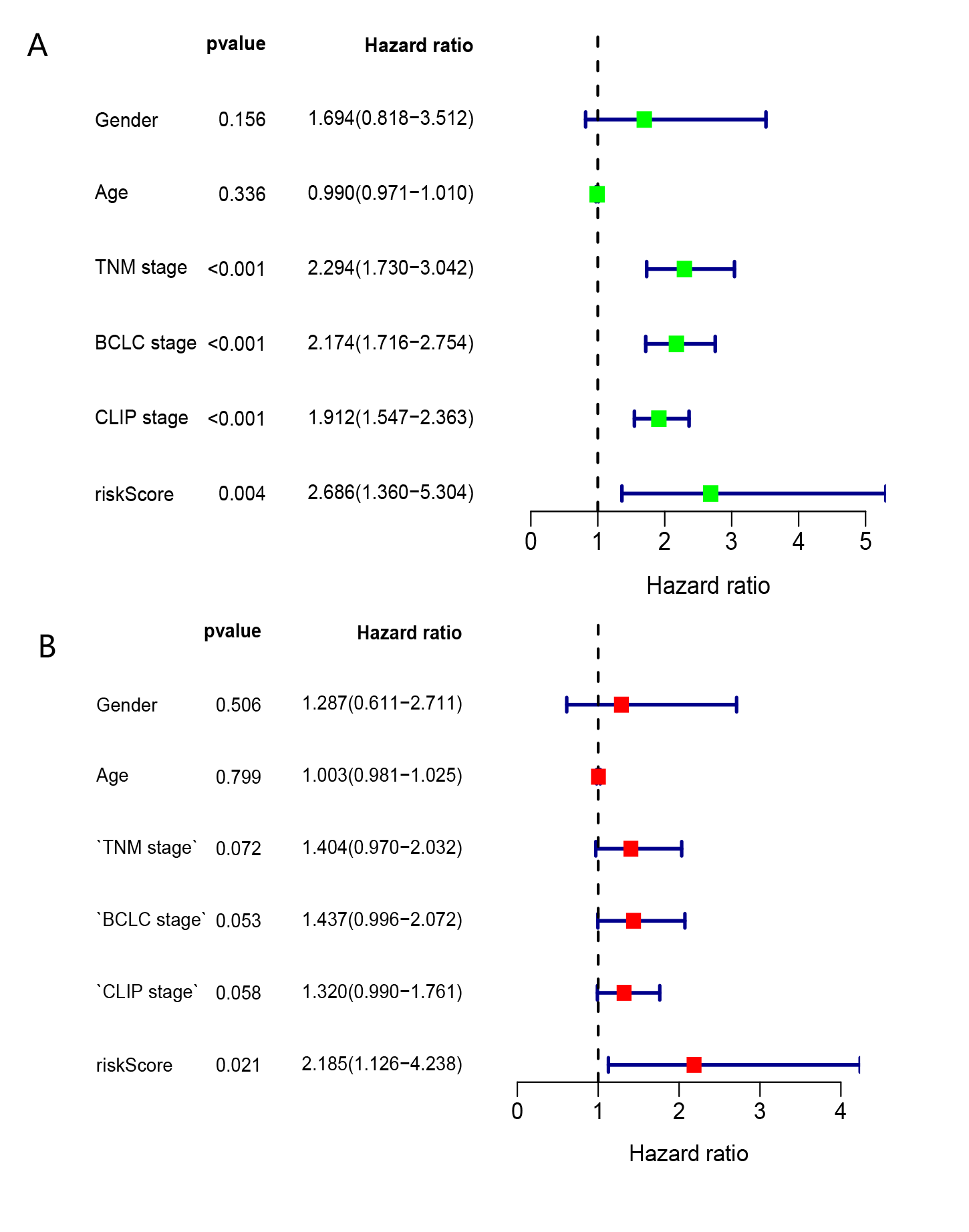

Supplement: Supplementary file 3 — Additional file 3. Univariate and Multivariate Cox analysis of risk score, gender, age, TNM- stage, CLIP-stage, and BCLC- stage in GSE14520 cohort. [file 12920_2023_1504_MOESM3_ESM.docx]
